# Correction: Deguelin inhibits growth and prolactin synthesis in prolactinomas by targeting the PI3K/AKT/CREB3L1 pathway and ornithine decarboxylase

**DOI:** 10.1038/s41401-026-01769-x

**Published:** 2026-02-23

**Authors:** Lei Gong, Chang-xiao-feng Liu, Jian-hua Cheng, Jing Guo, Bin Li, Hong-yun Wang, Meng Liu, Jia-lin Wang, Xue-jing Li, Qiu-yue Fang, Zhao-yi Yi, Chu-zhong Li, Ya-zhuo Zhang, Wei-yan Xie

**Affiliations:** 1 Beijing Neurosurgical Institute, Capital Medical University, Beijing, 100070 China; 2 Department of Neurosurgery, Beijing Tiantan Hospital Affiliated to Capital Medical University, Beijing, 100070 China; 3 Department of Neurosurgery, The Affiliated Hospital of Qingdao University, Qingdao, 266000 China; 4 Department of Neurosurgery, Peking University Third Hospital, Peking University, Beijing, 100191 China; 5 Department of Neurosurgery, Peking University People’s Hospital, Beijing, 100044 China; 6 Department of Neurosurgery, Sichuan Provincial People’s Hospital, University of Electronic Science and Technology of China, Chengdu, 610072 China

Correction to: *Acta Pharmacologica Sinica* 10.1038/s41401-025-01686-5, published online 03 November 2025

The authors regret to report errors in the paper published. We found unintentional misassembly of Western blot (WB) image in Fig. 4g. This wrong image is replaced by right ones of the same batch. However, these errors will not affect the correctness of the results of the paper.

The correct data were shown below:
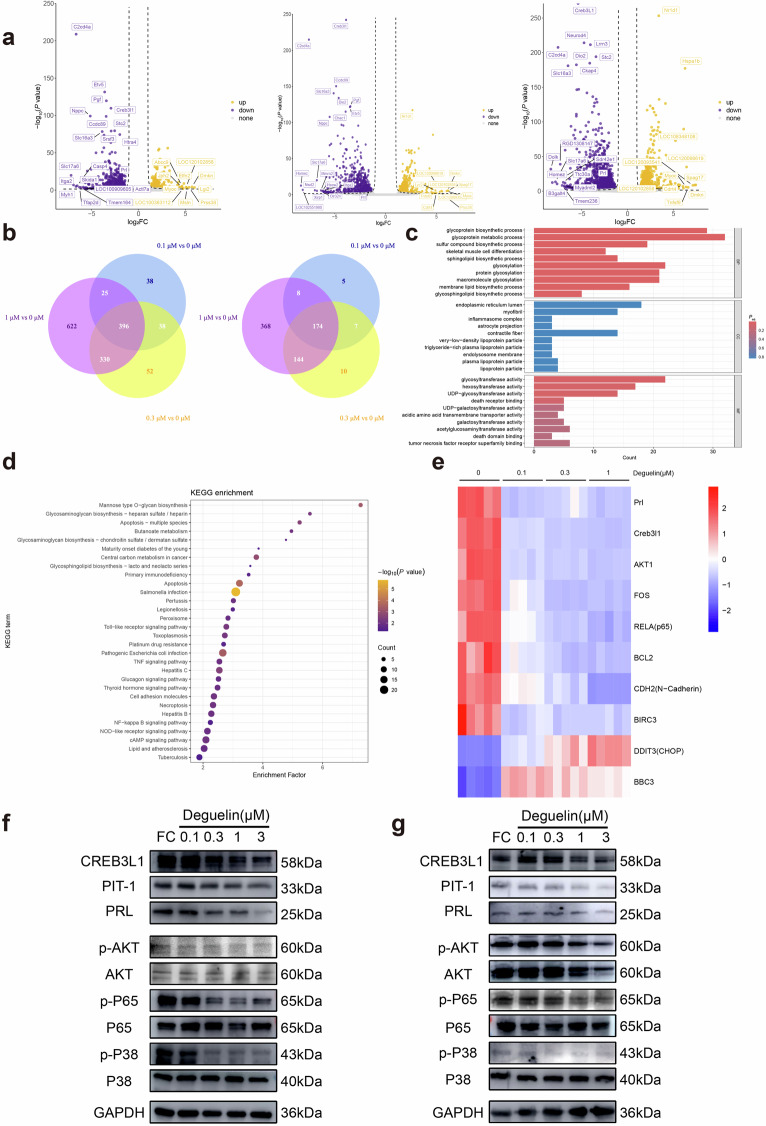
**Fig. 4 Transcriptome sequencing alterations in tumor-associated signaling pathways in GH3 cells after deguelin treatment**. **a** Volcano plot showing the inverse curve threshold line (threshold: *P*-value = 0.05, log2FoldChange = 1). The differential genes are located at the top left or top right of the curve. The top 10 upregulated genes (in descending order of log2FoldChange), the top 10 downregulated genes (in ascending order of log2FoldChange), and the top 10 differential genes ranked by ascending *P*-value are presented. **b** Venn diagram showing the intersection of three concentration downregulated genes (left) and upregulated genes (right) in significant differential genes. **c** The GO enrichment analysis of the downregulated significant differentially expressed genes. **d** KEGG pathway analysis of downregulated differentially expressed genes suggested enrichment of TNF and NF-κB pathway. **e** Heatmap showed expression of prolactin, TNF, and NF-κB pathway-associated gene between the groups. **f, g** Immunoblotting of lactogen-associated proteins with proteins from key nodes of the transcriptome enrichment pathway in deguelin-treated GH3 (**f**) or MMQ (**g**) cells for 48 h.

The original article has been corrected.

